# Amino acid supplementation in mitochondrial aminoacyl-tRNA synthetase defects: two case reports of tyrosine supplementation in *YARS2*-associated disease and a review of the literature

**DOI:** 10.3389/fped.2025.1699348

**Published:** 2025-12-01

**Authors:** Giulia Ferrera, Giorgia Segre, Eleonora Lamantea, Daniele Ghezzi, Marta Rivelli, Anna Ardissone

**Affiliations:** 1 Unit of Child Neurology, Fondazione IRCCS Istituto Neurologico “Carlo Besta”, Milan, Italy; 2 Ph.D. Program in Translational Medicine, University of Milan, Milan, Italy; 3 Unit of Medical Genetics and Neurogenetics, Mariani Centre for Paediatric Mitochondrial Disorders, Fondazione IRCCS Istituto Neurologico “Carlo Besta”, Milan, Italy; 4 Department of Pathophysiology and Transplantation, University of Milan, Milan, Italy; 5 Hospital Pharmacy, Fondazione IRCCS Istituto Neurologico “Carlo Besta”, Milan, Italy

**Keywords:** MLASA2, *YARS2*, aminoacyl-tRNA synthetase defect, *ARS2*, tyrosine, treatment, amino acids

## Abstract

**Background:**

Mitochondrial diseases (MDs) caused by pathogenic variants in aminoacyl-tRNA synthetase (*ARS*) genes, either cytosolic (*ARS1*) or mitochondrial (*ARS2*), are rare and clinically diverse. *YARS2* deficiency causes myopathy, lactic acidosis, and sideroblastic anemia (MLASA2). No treatments exist, although targeted amino acid (AA) supplementation could function as a possible therapy, as many *ARS* variants retain partial activity. While benefits have been reported in several *ARS1* disorders, evidence in *ARS2* diseases, including *YARS2* deficiency, remains limited.

**Methods:**

We report two siblings with genetically confirmed MLASA2 due to homozygous *YARS2* variants who received oral tyrosine for 12 months. Clinical, biochemical, cardiac, and thyroid safety assessments were performed at baseline and follow-up. Standardized measures tracked motor function, symptoms, and quality of life. A systematic review of AA supplementation in *ARS2* deficiencies was also conducted.

**Results:**

Tyrosine was well tolerated. The more severely affected sibling showed improvements in motor function, endurance, and quality of life, with modest prolongation of transfusion intervals. The milder sibling reported increased energy and functional gains. Cardiac function remained stable. Literature review revealed only five prior *ARS2* cases treated with AA supplementation, with variable outcomes.

**Conclusion:**

*YARS2-*related MLASA2 is a severe disorder associated with high morbidity and premature mortality. No spontaneous recovery has been reported, supporting tyrosine as the likely driver of observed improvements. No cardiac or thyroid toxicities were detected during treatment. Prior reports, although limited, support the feasibility of this treatment. Our findings suggest tyrosine is a promising candidate therapy in *YARS2* deficiency; larger multicenter studies are needed to validate our data.

## 1 Introduction

Mitochondrial diseases (MDs) are clinically and genetically heterogeneous disorders of energy metabolism across multiple organ systems (1). Among their genetic contributors, pathogenic variants in nuclear-encoded aminoacyl-tRNA synthetases (*ARS*s) represent a growing subgroup (2).

*ARS*s are enzymes that ensure accurate protein synthesis by attaching amino acids (AAs) to their cognate tRNAs. Each AA is paired with a dedicated enzyme, which functions in either the cytosol (cyt-ARS, encoded by *ARS1* genes) or mitochondria (mt-ARS, encoded by *ARS2* genes); some enzymes, including *GARS1* and *KARS1*, serve both compartments (2).

Mutations in *ARS2* genes cause variable phenotypes, often involving the central nervous system (CNS) (3, 4), although non-neurological phenotypes have been well-documented. Notably, biallelic *YARS2* variants cause myopathy, lactic acidosis, and sideroblastic anemia (MLASA2), a well-characterized syndrome (5, 6).

Therapies remain largely supportive; however, since many *ARS2* variants retain partial enzyme activity, targeted AA supplementation has been proposed as a potential treatment strategy (2). While this approach shows promise in ARS1 disorders (7), evidence supporting its application in mt-ARS deficiencies remains limited (8).

Here, we report two siblings with biallelic YARS2 mutations who showed clinical improvement with targeted tyrosine supplementation, together with a systematic review of mt-ARS disorders treated with their corresponding AA. We aim to provide preliminary evidence for the efficacy of tyrosine in *YARS2*-associated disease and to inform clinical management of other *ARS2*-related disorders.

## 2 Case descriptions and review of the literature

### 2.1 Case descriptions

We reviewed the clinical course of two siblings with genetically confirmed biallelic *YARS2*-associated MLASA2, diagnosed and followed at our institution. Clinical data—including family history, age, symptoms at disease onset, and longitudinal progression, instrumental, biochemical, and genetic tests performed over time—were retrospectively analyzed. As part of their clinical management, both patients initiated oral tyrosine supplementation (160 mg BID) alongside their established mitochondrial regimen. Given the experimental nature of the treatment and the theoretical risk of thyroid overstimulation, we opted for a conservative dosing strategy within a range known to be well tolerated and safe. Given the experimental nature of the treatment and the theoretical risk of thyroid overstimulation, we opted for a conservative dosing strategy within a range known to be well tolerated and safe (AA supplementation for malnutrition) (9), with no dose titration. The compound was prepared by the institutional pharmacy as a galenic powder.

Structured follow-up was conducted at baseline (T0) and 1, 2, 4, 6, 8, and 12 months after treatment initiation (T1–T6). At each visit, routine labs were obtained (blood gas, blood count, liver enzymes, creatine phosphokinase (CPK), coagulation, electrolytes, glucose, renal function). Cardiac safety was monitored via EKGs; thyroid function [thyroid-stimulating hormone (TSH), free triiodothyronine (fT3), free thyroxine (fT4)] was assessed at every visit due to the theoretical risk of tyrosine excess. Plasma and urinary tyrosine levels were measured during the first 6 months.

A pediatric neurologist conducted neurologic evaluations, monitored adherence and adverse events, and administered standardized outcome measures. These included the Newcastle Pediatric Mitochondrial Disease Scale (NPMDS) (10), which assesses disease burden and functional impairment in children with MDs across neurological, systemic, and functional domains; the Clinical Global Impression (CGI), a clinician-rated scale providing a global assessment of illness severity and change over time (11); the North Star Ambulatory Assessment (NSAA), a functional motor scale designed to evaluate ambulatory abilities in children with neuromuscular conditions (12); the 6-Minute Walk Test (6MWT), measuring the distance a patient can walk in 6 min to reflect overall functional mobility and endurance (13); and the Pediatric Quality of Life Inventory (PedsQL), assessing health-related quality of life in children across physical, emotional, social, and school functioning (14).

**Figure 1:**
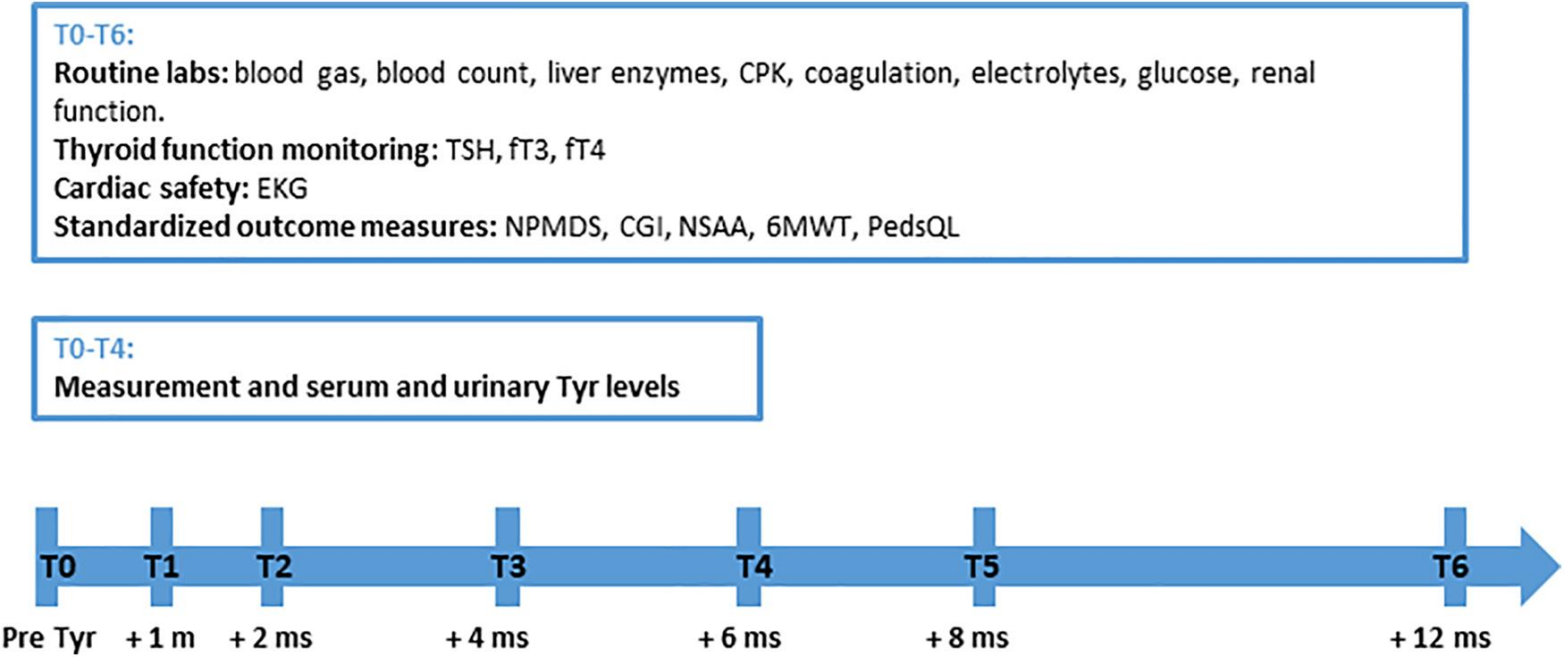
Study methodology. 6MWT, 6-Minute Walk Test; CGI, Clinical Global Impression; NMPDS, Newcastle Pediatric Mitochondrial Disease Scale; NSAA, North Star Ambulatory Assessment; PedsQL, Pediatric Quality of Life Inventory; Tyr, tyrosine.

Figure 1 depicts the study methodology.

The study followed the Declaration of Helsinki, using anonymized routine clinical data in accordance with institutional regulations. Tyrosine was prescribed as a compounded, named-patient preparation under Italian and EU rare disease regulations, with approval from the Lombardy Coordination Center for Rare Diseases. Ethics committee approval was not required per institutional policies. Parental consent was obtained for off-label treatment and data use (collection and publication).

#### 2.1.1 Patient 1

This previously reported male (5), first child of non-consanguineous parents, presented at 2 months with anemia, neutropenia, and hyperlactatemia, requiring transfusions until 14 months. Subsequent hematologic normalization was spontaneous. At 1 year 11 months, he was diagnosed with MLASA2 following identification of a homozygous *YARS2* c.933C>G (p.Asp311Glu) variant. Neurological evaluation at diagnosis was normal.

At age 6, fatigue and mild proximal lower limb weakness developed. Anemia recurred at 9.5 years, but did not initially require regular transfusions. Alternating esophoria emerged at 10 years. Bone marrow aspiration at 11 confirmed sideroblastic anemia, and by age 12, transfusion dependence resumed (∼every 10 days). At age 13, MRI revealed hepatic iron overload, treated with chelation. The same year, polysomnography showed obstructive sleep apnea with hypercapnia, but non-invasive ventilation was not tolerated by the patient. Compensated adrenal insufficiency was diagnosed, managed with stress-dose steroids (0.6 mg/kg) in cases of acute illnesses. At age 15, cardiac studies showed upper-normal left ventricular thickness; repeat MRI confirmed stable hepatic iron.

At 15 years, 7 months (T0), neurological examination showed alternating exotropia, hypotonia, and significant proximal lower limb weakness. Gait was waddling; ambulation was <100 m unaided. He could not run and needed knee support to rise from the floor. Mingazzini test on the upper and lower limbs was sustained for over 40 and 8 s, respectively.

Tyrosine (160 mg BID) was added to ongoing therapy (ubidecarenone, riboflavin, folinic acid, sodium bicarbonate, iron chelation) with good compliance. Baseline labs showed cytopenia (RBC 2.8 × 10^12^/L, WBC 2.2 × 10^9^/L, Hb 8.4 g/dL) and subclinical hypothyroidism (TSH 3.67 mIU/L; low-normal fT4). Hemoglobin remained stable (∼8.5 g/dL) during follow-up; subclinical hypothyroidism normalized. Follow-up EKGs remained normal; a follow-up cardiac ultrasound, after 12 months of supplementation, showed stable findings.

Transfusion frequency stayed bimonthly, although on two occasions intervals extended to 20–24 days (after 4 and 5 months, respectively). Plasma and urinary tyrosine levels remained within normal range.

**Figure 2:**
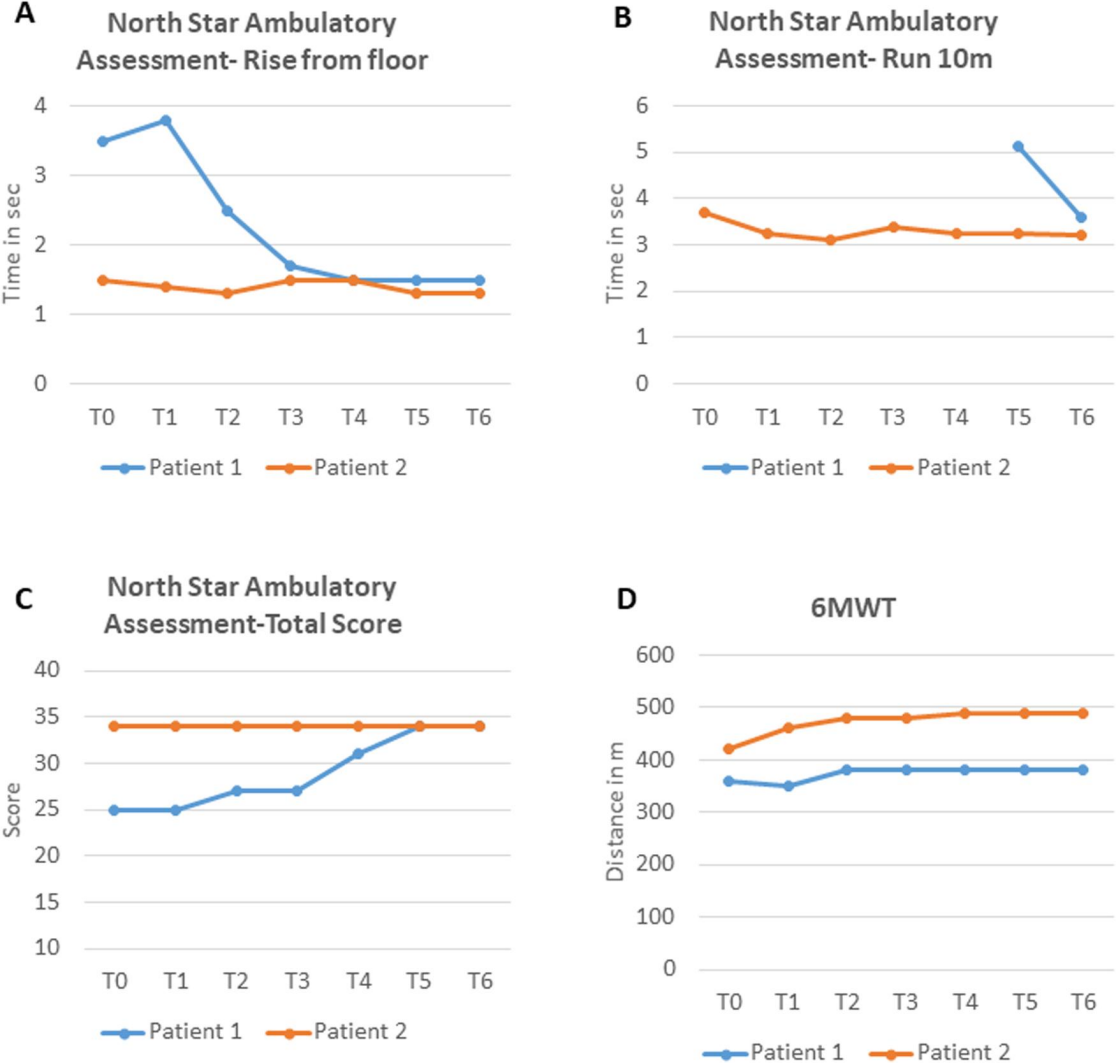
**(A–C)** NSAA subscores and total score for both patients at baseline and during treatment; **(D)** 6MWT time for both patients at baseline and during treatment. NSAA, North Star Ambulatory Assessment; 6MWT, 6-Minute Walk Test; sec, seconds; m, meters.

Motor function improved. After 12 months, gait was better, and he could climb stairs alternating steps, rise from the floor in 1.5 s (vs. 3.5 at T0) (Figure 2A), and run short distances (10 m in 3.6 s) (Figure 2B). Mingazzini tests on the upper and lower limbs both exceeded 40 s. No new neuromuscular signs emerged. NPMDS scores also remained stable, confirming the stability of the patient's MD. NSAA scores improved from 25/34 (T0) to 34/34 by T5, remaining stable thereafter (Figure 2C). 6MWT increased modestly: 360–380 m at T2 (Figure 2D), stabilizing thereafter.

**Figure 3:**
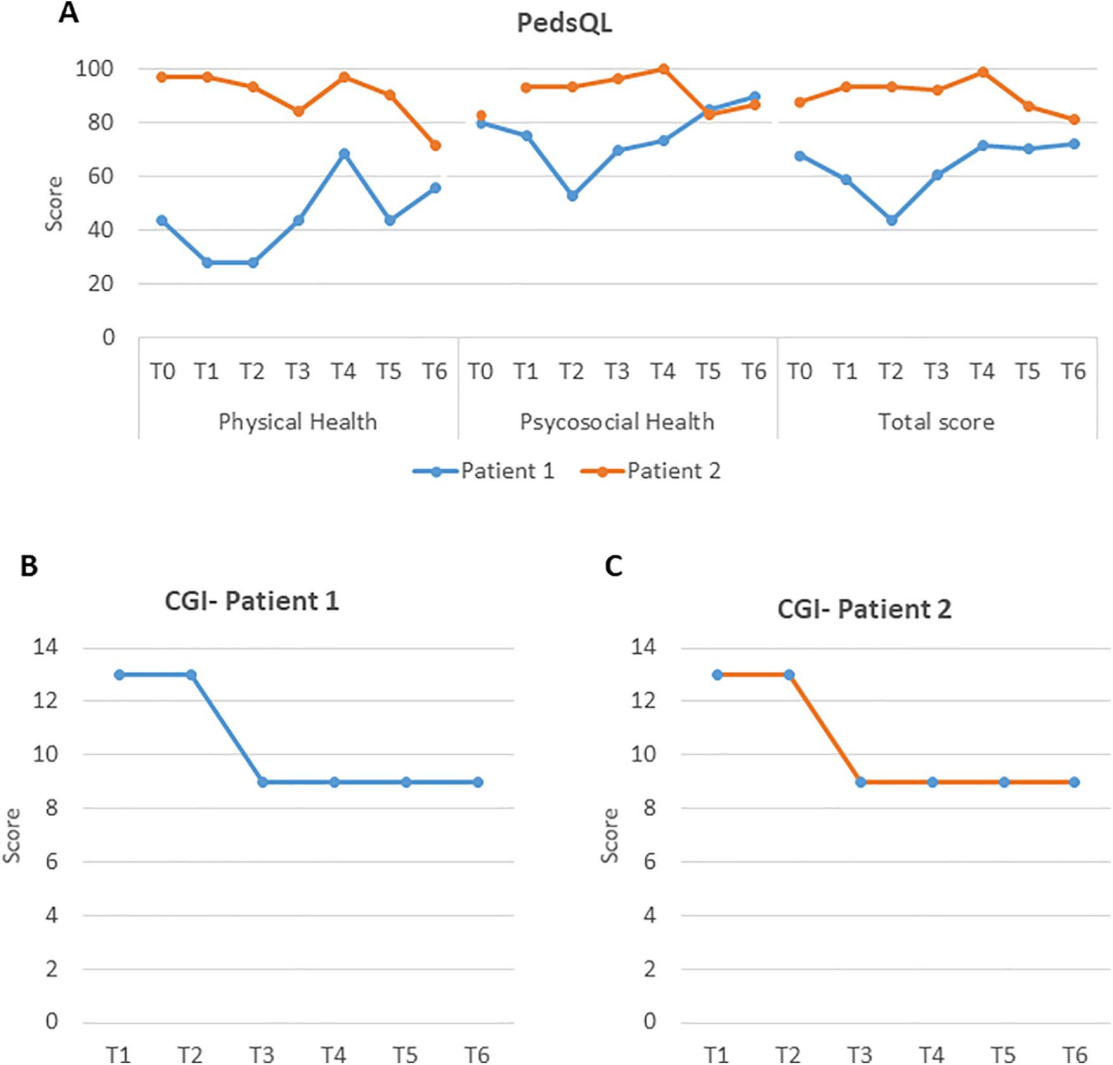
**(A)** PedsQL scores for both patients at baseline and during treatment. **(B)** CGI scores for Patient 1 at baseline and during treatment. **(C)** CGI scores for Patient 2 at baseline and during treatment. PedsQL, Pediatric Quality of Life Inventory; CGI, Clinical Global Impression.

Subjective stability was reported up to T3. From T4, the patient and his family reported less fatigue and greater energy, corroborated by objective findings. Specifically, PedsQL scores increased from T4 onward, especially in physical functioning, and then plateaued (Figure 3A). Finally, the CGI scale also captured an overall clinical improvement with no side effects (score of 9), first noted at T3 and then maintained (Figure 3B).

#### 2.1.2 Patient 2

Patient 1's younger sister by 5 years, also previously reported (5), was diagnosed with MLASA2 on day 7 of life; neurological exam at diagnosis was normal. She had mild anemia and lactic acidosis from 2 months, requiring six transfusions (last at 9 months), with stable hemoglobin thereafter. Psychomotor development was normal. She showed transient biventricular hypertrophy at 9 months, resolving by 30 months, with a brief recurrence at 4.5 years that regressed fully by age 11. For 4.5 years, she reported post-exertional fatigue with minimal lifestyle impact. At 8 years, precocious puberty was diagnosed; brain MRI was normal.

At 10 years, 7 months (T0), tyrosine (160 mg BID) was initiated with ongoing supplements (ubidecarenone, riboflavin, sodium bicarbonate). Neurological exam pre-supplementation was unremarkable. Baseline bloodwork showed mild microcytic anemia (Hb ∼ 10.5 g/dL), neutropenia (WBC ∼ 4.67 × 10^9^/L), and subclinical hypothyroidism (TSH 4.99 μIU/mL fT3 and fT4 within normal values), all stable during follow-up. Tyrosine levels remained within range, with one mild transient plasmatic elevation (97 μmol/L, reference values 40–92 μmol/L).

Follow-up EKGs remained normal; a follow-up cardiac ultrasound, after 12 months of supplementation, confirmed normal findings.

Objective assessments showed motor improvements over time. NSAA was maximal at T0 (34/34), but timed subtests improved, including the 10 m run (3.71–3.21 s) and rise from floor (1.5–1.3 s) (Figures 2A–C). 6MWT increased from 420 m (T0) to 490 m (T4) and then stabilized (Figure 2D).

Subjective improvements were also noted from T3 onwards—when the patient described feeling more energetic, with improved exercise tolerance and an overall sense of well-being—persisting throughout follow-up. Neurological exam remained normal. PedsQL scores fluctuated throughout follow-up, and the total score at T6 was slightly lower than at T0 (81.5 vs. 88) (Figure 3A). However, scales assessing overall disease status reflected clinical improvement. CGI captured an overall clinical improvement with no side effects (score of 9), first noted at T3 and maintained subsequently (Figure 3C). NPMDS remained stable.

### 2.2 Literature review

We searched PubMed for English-language case reports of patients with pathogenic variants in any of the 17 *ARS2* genes (*AARS2*, *RARS2*, *NARS2*, *DARS2*, *CARS2*, *EARS2*, *HARS2*, *IARS2*, *LARS2*, *KARS1*, *MARS2*, *FARS2*, *PARS2*, *SARS2*, *TARS2*, *WARS2*, *YARS2*, *VARS2*) treated with their cognate AA. Abstracts, conference proceedings, non-English, and non-clinical studies were excluded.

As of August 2025, AA supplementation has been reported in five patients with two mt-ARS deficiencies, across two publications (8, 15). Treatment protocols, dosages, and outcomes varied between individuals (see Table 1 for a detailed summary).

**Table 1:** Clinical, genetic, and treatment data for all patients identified through our literature review.

Ref.	Specific *ARS2* deficiency	Phenotype	Patient age at T0	AA supplementation; dosage	Treatment protocol	Follow-up time	Side effects	Effects of treatment
Shen (8)	*RARS2* deficiency	Epilepsy; no other clinical information available	NA	Arginine; 150–350 mg/kg twice a day	NA	NA	None reported	No improvement on seizures; improved EEG. Child was described as more energetic
Shen (8)	*RARS2* deficiency	Epilepsy; no other clinical information available	NA	Arginine; 150–350 mg/kg twice a day	NA	NA	None reported	No improvement on seizures; improved EEG. Child was described as more energetic
Shen (8)	*RARS2* deficiency	Epilepsy; no other clinical information available	NA	Arginine; 150–350 mg/kg twice a day	NA	NA	None reported	No improvement on seizures; improved EEG. Child was described as more energetic
Shen (8)	*RARS2* deficiency	Epilepsy; no other clinical information available	NA	Arginine; 150–350 mg/kg twice a day	NA	NA	None reported	No improvement on seizures; improved EEG. Child was described as more energetic
Oswald et al. (15)	*FARS2* deficiency	Reduced muscular strength; truncal muscular hypotonia with hypertonia of the lower extremities, cerebellar ataxia (gait ataxia, intention tremor, and dysarthria)	3 years	Phenylalanine; 150 mg/die	*T0 (baseline):* physical examination, MABC-2, DGI, GMFM-66, PedsQL, laboratory testing (plasma amino acid profiling, complete blood count, liver enzymes, kidney function).	10.5 months	None reported	Improvement in all subtests of the test battery. Clinically:
					*T1 (T0* *+* *21 weeks):* physical examination, MABC-2, DGI, GMFM-66, PedsQL. Phenylalanine was then suspended			*T1:* improvement of cerebellar signs (speech was more fluid, gait was more stable, tremor was reduced)
					*T2 (T0* *+* *29 weeks):* physical examination, MABC-2, DGI, GMFM-66, PedsQL. Phenylalanine was restarted			*T2*: worsening of cerebellar signs (speech was dysarthric, gait was broader, and she was more unstable)
					*T3 (T0* *+* *42 weeks):* physical examination, MABC-2, DGI, GMFM-66, PedsQL.			*T3*: improvement of cerebellar signs (gait was improved, speech was rated age-adequate for clarity and fluidity. Generally described as more energetic
This paper, Patient 1	*YARS2* deficiency	MLASA2	15 years, 7 months	Tyrosine; 160 mg/ twice a day	*T0 (baseline), T1 (T0* *+* *1 month), T2 (T0* *+* *2 months), T3 (T0* *+* *4 months), T4 (T0* *+* *6 months), T5 (T0* *+* *8 months), T6 (T0* *+* *12 months):* laboratory testing (arterial blood gas, complete blood count, liver enzymes, coagulation profile, serum electrolytes, glucose, renal and thyroid function); neurological examination, EKG; NPMDS, CGI, NSAA, 6MWT, PedsQL	14 months	None reported	Improvement in all subtests of the test battery
					*T0, T1, T2, T3, T4:* tyrosine levels in blood and urine			Clinically, from *T4* onwards, the patients reported less fatigue and greater energy. At *T6*, gait was better, and he could run short distances, climb stairs alternating steps, and rise from the floor faster
This paper, Patient 2	*YARS2* deficiency	MLASA2	10 years, 7 months	Tyrosine; 160 mg/ twice a day	*T0 (baseline), T1 (T0* *+* *1 month), T2 (T0* *+* *2 months), T3 (T0* *+* *4 months), T4 (T0* *+* *6 months), T5 (T0* *+* *8 months), T6 (T0* *+* *12 months):* laboratory testing (arterial blood gas, complete blood count, liver enzymes, coagulation profile, serum electrolytes, glucose, renal and thyroid function); neurological examination, EKG; NPMDS, CGI, NSAA, 6MWT, PedsQL	14 months	None reported	Improvement in all subtests of the test battery except PedsQL; NSAA already was at maximum score at *T0*
					*T0, T1, T2, T3, T4:* tyrosine levels in blood and urine			Clinically, the patient reported feeling more energetic, with improved exercise tolerance and an overall sense of well-being

6MWT, 6-Minute Walk Test; CGI, Clinical Global Impression; DGI, Dynamic Gait Index; EEG, electroencephalogram; EKG, electrocardiogram; GMFM-66, Gross Motor Function Measure—66-Item Version; MABC-2, Movement Assessment Battery for Children—Second Edition; NA, not available; NSAA, North Star Ambulatory Assessment; PedsQL, Pediatric Quality of Life Inventory; T0, baseline (pre-treatment).

In *FARS2* deficiency, a 3-year-old with hypotonia, hypertonia of the lower limbs, and cerebellar signs received phenylalanine (150 mg/day) in an n-of-1 trial with serial evaluations over 10.5 months, including an 8-week withdrawal period (15). Treatment was well tolerated, and improvements were observed across all motor and developmental assessment subtests. Clinically, at the last follow-up, the child showed improved speech clarity, less ataxic gait, and a new ability to alternate feet when climbing stairs.

In *RARS2* deficiency, four patients with epilepsy were treated with arginine (150–350 mg/kg, twice daily) (8). Follow-up duration and pre-supplementation clinical phenotyping for involved patients were not reported. Although seizure frequency did not improve, electroencephalogram (EEG) findings improved in all cases, and families reported increased energy. No side effects occurred.

## 3 Discussion

Initially considered rare, mutations in *ARS2* genes have, over the past two decades, emerged as significant contributors to pediatric MDs (2). Pathogenic variants have been identified in all 17 *ARS2* genes, each linked to distinct but often overlapping phenotypes. Despite their ubiquitous expression, mt-ARS deficiencies predominantly affect the CNS, presenting as leukodystrophies (*AARS2*, *DARS2*, *EARS2*, and occasionally *LARS2*), Leigh syndrome (*MARS2*, *PARS2*, *NARS2*, *IARS2*), sensorineural hearing loss with ovarian failure (*LARS2*, *HARS2*), epilepsy (*VARS2*, *FARS2*, *RARS2*, *CARS2*), and extrapyramidal syndromes (*WARS2*, *CARS2*) (2, 16, 17). Only two mt-ARS deficiencies are primarily associated with extra-CNS manifestations: *SARS2*, linked to renal disease, and *YARS2*, which causes the MLASA2 phenotype (2, 5, 16, 18).

Due to the rarity of *YARS2*-associated disease, its natural history remains incompletely defined. Approximately 40 cases have been reported (5, 18–25), typically presenting in early infancy/childhood (median onset 48 months), although rarer adolescent- and adult-onset cases have been reported (10% of cases) (5, 18–25). Most patients first show transfusion-dependent anemia (60%), with myopathy either concurrent (30%) or developing later (70%) (5, 18–25). In time, the clinical spectrum ranges from isolated sideroblastic anemia (30% of cases) to full-blown MLASA2 (approximately 60%), with intermediate forms in the remainder (5, 18–25). Rare asymptomatic cases with biallelic *YARS2* mutations (*n* = 2) further underscore the phenotypic variability (18). The disease is associated with high morbidity: transfusion-dependent anemia is seen in ∼75% of cases, progressive myopathy leading to loss of ambulation, and hypertrophic cardiomyopathy in ∼33%. Approximately 25% of cases die prematurely, often from cardiac or respiratory failure (5, 18–25).

The two patients showed phenotypes typical of *YARS2*-associated disease but with striking variability. Patient 1 developed early transfusion-dependent sideroblastic anemia, progressive childhood-onset myopathy, and later compensated cardiomyopathy. In contrast, Patient 2 required transfusions only in infancy and showed no myopathy or cardiomyopathy. These cases highlight the broad clinical spectrum of *YARS2* disease, even within a single family (18).

We report the beneficial effects of oral L-tyrosine supplementation in two patients with homozygous *YARS2* variants. Over 12 months, both showed notable improvement, including reduced myopathic symptoms and enhanced quality of life, most evident in the more symptomatic patient. No adverse effects were detected. Patient 1 gained endurance, acquired new motor skills, and improved performance across standardized scales. Quality of life, after an initial slight decline due to emotional difficulties, increased from T3 onwards. Patient 2, largely asymptomatic at baseline, reported increased energy and stamina. Importantly, no new symptoms emerged in either patient, and clinical stability was confirmed via unchanged NPMDS scores. Patient 2's quality-of-life scores did fluctuate, but likely reflected emotional and school-related challenges during follow-up rather than an increased disease burden.

To our knowledge, spontaneous improvement of myopathy has not been reported in *YARS2* disease (5, 18–25), where the course is usually stable or progressive. Thus, the improvements observed here, especially in Patient 1, are more likely attributable to a true therapeutic effect of L-tyrosine than to natural variability in disease progression. Moreover, Patient 1 also twice extended transfusion intervals from the usual 10–20–24 days. Typically transfused every 10 days, he was able on two occasions to extend this to 20 and 24 days. While promising, transfusion needs can fluctuate in *YARS2*-associated disease even without treatment (6). Further data are needed to clarify any hematologic benefit of tyrosine supplementation.

No adverse effects occurred during supplementation. Thyroid function, which can theoretically be affected by excess tyrosine, remained within normal limits, and both EKGs and cardiac ultrasounds revealed no abnormalities. Our data highlight a favorable safety profile of treatment with tyrosine in *YARS2*-associated disease.

The tyrosine dosage used in our two patients was based on previously established safe levels, particularly those used for malnutrition (9). This conservative approach was chosen due to the pilot nature of the study and the limited data available on the safety and tolerability of tyrosine supplementation in this context. Future studies should investigate the effect of a higher dosage of tyrosine supplementation and the potential for a dose-dependent effect.

Our review identified two reports of AA supplementation in *ARS2* defects. In *FARS2* deficiency, a 42-week trial of phenylalanine produced substantial gains in motor skills, postural stability, and quality of life (15). Conversely, in a cohort of four patients with *RARS2* pathogenic variants, only minor improvements in EEG and subjective reports of increased energy were noted (8). These patients also had poorly characterized baseline phenotypes but were described as harboring epilepsy, which is usually severe and debilitating in *RARS2*-associated disease. These findings suggest that AA supplementation may be less effective when initiated in advanced stages of disease.

This pattern mirrors *ARS1* disorders, where responses in the 21 patients described to date are gene- and context-specific (7). Methionine supplementation in over 10 patients with *MARS1* deficiency has yielded consistent clinical benefit, including resolution of pulmonary disease, improved growth and feeding, and prevention of recurrence posttransplant (7). Smaller cohorts have also shown potential benefits from isoleucine (*IARS1*) and leucine (*LARS1*) supplementation, with observed improvements in growth, liver function, and infection resistance (7). In contrast, lysine (*KARS1*) and phenylalanine (*FARSA/B*) yielded variable or transient responses, while glutamine (*QARS1*) and tyrosine (*YARS1*) appeared ineffective (7). Notably, in the latter cases, patients exhibited extremely severe phenotypes, including profound psychomotor delay and progressive multiorgan failure. As with *ARS2* disorders, these findings suggest that the severity and timing of intervention may critically influence therapeutic efficacy.

A strength of our study is the systematic use of standardized, validated clinical scales to monitor therapeutic response over time. In the context of rare disorders such as *ARS2* deficiencies, where clinical heterogeneity and small sample sizes pose significant challenges, the implementation of structured protocols is essential. We employed multiple outcome measures (NPMDS, NSAA, 6MWT, CGI, and PedsQL) to capture both objective and subjective changes in motor function, global health status, and quality of life. This multimodal, longitudinal approach not only increases the reliability of our findings but also sets a precedent for future studies seeking to evaluate therapeutic efficacy in a reproducible and comparable manner.

Our study also has some limitations. Most notably, the lack of a control group and the small sample size restrict the generalizability of our findings. The follow-up period, while sufficient to detect short-term changes, may not fully capture the long-term safety or sustained efficacy of tyrosine supplementation. Nonetheless, our results lay the groundwork for a safe, effective treatment for these rare and debilitating disorders. Future research should aim to address these gaps through randomized controlled trials in larger, ideally multicenter cohorts, with extended longitudinal follow-up.

## Data Availability

The datasets presented in this article are not readily available because of ethical and privacy restrictions. Requests to access the datasets should be directed to the corresponding author/s.
